# Growth analysis among adolescents with moderate-to-severe atopic dermatitis receiving upadacitinib in combination with topical corticosteroids in Japan: A case study series from a phase 3, randomized, controlled trial (Rising Up)

**DOI:** 10.1016/j.waojou.2022.100678

**Published:** 2022-09-13

**Authors:** Toshiaki Tanaka, Takuya Sasaki, Kimitoshi Ikeda, Jianzhong Liu, Allan R. Tenorio, Yukihiro Ohya

**Affiliations:** a Tanaka Growth Clinic, Tokyo, Japan; b AbbVie GK, Tokyo, Japan; c AbbVie Inc., North Chicago, IL, United States; d National Center for Child Health and Development, Tokyo, Japan

**Keywords:** Growth, Janus kinase inhibitors, Reference growth curves, Upadacitinib, Atopic dermatitis

## Abstract

**Background:**

Treatment options for adolescents with moderate-to-severe atopic dermatitis (AD) are limited. Oral corticosteroid therapies are used to treat children and adolescents with moderate-to-severe AD; however, long-term use is not recommended because of potential growth impairment. Upadacitinib, an oral Janus kinase inhibitor, is approved to treat moderate-to-severe AD in the United States, Japan, and Europe. To investigate potential effects of upadacitinib on growth, we analyzed height and height velocity in 6 adolescent patients in the phase 3 Rising Up study who were in the decline phase of pubertal growth at the time of study entry.

**Methods:**

The randomized, double-blind, Rising Up (NCT03661138) study compared upadacitinib plus topical corticosteroids (TCS) to placebo plus TCS in adolescents and adults with moderate-to-severe AD in Japan. Eligible adolescents (aged 12–17 years) were randomized 1:1:1 to receive once-daily upadacitinib 15 mg, upadacitinib 30 mg, or placebo in combination with TCS for 16 weeks. After 16 weeks, patients randomized to receive placebo were rerandomized 1:1 to receive upadacitinib 15 mg or upadacitinib 30 mg. Historical height measurements were obtained when available. Individual growth and growth velocity curves were compared with standard curves for Japanese youths. This non-prespecified analysis used 52-week data.

**Results:**

Of the 29 adolescents enrolled, 6 were in the decline phase of pubertal growth at enrollment. Growth curves and growth velocity curves for these 6 patients remained within the normal range for Japanese adolescents throughout the study. Biomarkers of bone metabolism generally remained stable over the course of the study. No musculoskeletal adverse events were reported.

**Conclusions:**

No cases suggested that upadacitinib negatively affected adolescent growth. Ongoing studies will continue to assess height and adverse effects related to bone growth to further inform on this patient group.

**Trial registration:**

ClinicalTrials.gov Identifier NCT03661138.

## Introduction

Atopic dermatitis (AD) is the most common inflammatory skin disorder, with a prevalence of 2–10% among adults and 15–30% in children, and an increasing incidence in industrialized countries in recent decades.^1^^,^^2^ In Japan, a survey of 48,702 children and adolescents conducted by the Ministry of Health, Labor, and Welfare found 10.6% and 8.2% rates of AD among those patients aged 12–13 years and 18 years, respectively.^3^ It is estimated that in as many as 50% of school-aged children and adolescents who are diagnosed with AD, the disease persists well into adulthood.^4^ Pruritis, the hallmark of AD, and skin pain affect sleep and school or work performance and can compromise patients’ quality of life.^5^, ^6^, ^7^ Furthermore, a pooled analysis of 9 US population–based studies suggests that children and adolescents with severe eczema accompanied by insufficient sleep may have vertical growth impairment.^8^

Topical corticosteroids (TCS) and emollients are the backbone of AD treatment. Treatment options for patients with moderate-to-severe AD not sufficiently controlled by TCS are limited. The older immunosuppressant therapies (eg, oral corticosteroids, cyclosporin A) are typically limited to short-term use due to side effects,^9^^,^^10^ may not be approved for use in AD^11^ and/or may lack robust evidence of efficacy and safety in patients with AD.^9^ In Japan, dupilumab and baricitinib, the relatively newer advanced systemic therapies, are only approved to treat moderate-to-severe AD in adults who are candidates for systemic therapy.^12^^,^^13^ Oral corticosteroid therapies, therefore, have been used in children and adolescents in Japan who have moderate-to-severe AD and who are not responding to TCS. Long-term use of oral corticosteroids, however, is not recommended due to side effects to which younger patients are more susceptible.^14^^,^^15^ Prolonged exposure to oral corticosteroids is associated with adrenal insufficiency and affects bone turnover, which may result in growth impairment.^16^^,^^17^

Although there is a major need for oral therapies for adolescents with AD who have not responded adequately to their current treatments, until recently there was no approved systemic treatment with sufficient efficacy and tolerability for long-term use. Long-term treatment options are especially needed for patients with severe AD during puberty. These patients are often hospitalized to administer topical anti-inflammatory agents, but this can be challenging due to social and educational considerations (eg, the need to prepare for entrance examinations, which is common in Japanese children at this age). An appropriate systemic treatment allowing for skin clearance and itch reduction while maintaining an acceptable safety profile in this population therefore constitutes a major unmet need.

Signaling via the Janus kinas (JAK)-signal transducer and activator of transcription-mediated pathway plays a key role in the pathophysiology of AD^18^ Upadacitinib is a once-daily, orally administered JAK inhibitor with greater potency for JAK1 than for other JAK family members.^19^ In patients aged 12 years and older with moderate-to-severe AD, once-daily, orally administered upadacitinib (15 mg or 30 mg) with (AD Up study [NCT03569318]) or without TCS (Measure Up 1 study [NCT03569293], Measure Up 2 study [NCT03607422]) was more effective than placebo during 3 large, phase 3 randomized, double-blind, placebo-controlled studies. Results from these studies as well as the Rising Up study (NCT03661138), which compared upadacitinib combined with TCS to placebo combined with TCS in Japan, showed that upadacitinib 15 mg and 30 mg were well tolerated, with a safety profile similar to that observed in studies in rheumatoid arthritis; no new important safety signals were reported.^19^, ^20^, ^21^

Upadacitinib is approved in the United States, Japan, Europe, and other countries for adults and adolescents with moderate-to-severe AD.^22^, ^23^, ^24^ Another JAK1 inhibitor, abrocitinib, was recently approved in Japan for adults and adolescents with moderate-to-severe AD^25^; however, in view of the microscopic findings related to growth observed in toxicity studies in juvenile rats, the European Medical Agency did not approve its use in adolescents.^26^ In contrast, in a toxicity study of upadacitinib using juvenile rats, no microscopic or macroscopic findings related to bone abnormalities were observed.^27^ As evidence suggests that the JAK-signal transducer and activator of transcription-mediated signaling pathway plays an important role in bone development,^28^ it is important to further investigate the clinical impact of JAK inhibitors on growth. Given the limited information available on the role of JAK inhibitors in bone growth, we performed a post hoc longitudinal examination of the long-term effects of upadacitinib on growth in 6 adolescents enrolled in the Rising Up study^21^ who were in the decline phase of pubertal growth at the time of study entry. In the Rising Up study, pre-enrollment height data from school and medical records were collected when available, and adolescent patient height was monitored over the course of the study.^21^

## Methods

### Patients and study design

Detailed methods used in the Rising Up (NCT03661138) study were previously reported.^21^ Briefly, patients in Japan (aged 12–75 years) with moderate-to-severe AD who were candidates for systemic therapy and had a history of inadequate response to topical or systemic AD treatments within the previous 6 months were eligible to participate. Eligible adolescent patients (aged 12–17 years) had weight ≥40 kg at baseline; patients with prior exposure to JAK inhibitors or dupilumab were excluded from participation. Patients were randomized 1:1:1 to receive once-daily upadacitinib 15 mg, upadacitinib 30 mg, or placebo, in combination with TCS for 16 weeks. After 16 weeks, patients who had been randomized to receive placebo were rerandomized 1:1 to receive once-daily upadacitinib 15 mg or upadacitinib 30 mg. Patients initially randomized to receive upadacitinib 15 mg or 30 mg continued treatment as originally assigned. The study was conducted in accordance with Good Clinical Practice Guidelines as defined by the International Conference on Harmonisation and the Declaration of Helsinki. The protocol was approved by institutional review boards at the study centers, and all parents or legal guardians of adolescent patients provided written informed consent. Patient anonymity was preserved using methods approved by the institutional review boards at all 42 participating clinical trial sites.

### Assessments

For adolescent patients, height measurements obtained before the first dose of study drug were collected when available from medical charts or school records, and height was measured without shoes at screening; baseline; and at weeks 8, 16, 24, 32, 40, and 52. Results from clinical laboratory evaluations, which included analysis of alkaline phosphatase, calcium, and phosphate concentrations in serum, were assessed at baseline; screening; weeks 2, 4, 8, 12, 16, 20, 32; visits every 12 weeks from weeks 64 through 160; unscheduled visits for rescue treatment; and at premature discontinuation from the study. Eczema Area and Severity Index (EASI) score and percent change in EASI from baseline were assessed at all visits. Safety evaluations were conducted at each study visit. The present analysis is based on data acquired when the last patient had completed the week 52 study visit. Growth and growth velocity curves for each patient were superimposed on the Japanese standard growth chart^29^ and growth velocity chart,^30^ and evaluated by a pediatric endocrinologist (Toshiaki Tanaka, MD).

### Statistical analysis

Results were reported descriptively for all patients receiving study treatment. Analysis of adolescent growth was post hoc, and no statistical testing was performed.

## Results

Of the 272 patients enrolled in the Rising Up study, 29 (11%) were adolescents, of whom 22 were males and 7 were females. Eighteen male and 5 female adolescent patients had already reached their adult height at the time of study entry or shortly after the study entry according to the evaluation of their growth curves. Six adolescent patients (4 males and 2 females) were in the decline phase of pubertal growth at the time of study entry. At baseline, these 6 patients’ ages ranged from 13 to 16 years, their height measurements ranged from 146.8 to 165.7 cm, and all were Tanner stage II or III. EASI scores at baseline ranged from 20.2 to 38.4, and all patients had received prior TCS therapy.

**Fig. 1:**
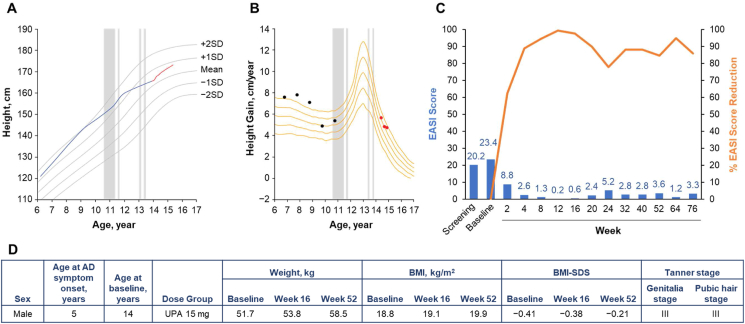
Patient 1 (male, aged 14 years) in the upadacitinib 15-mg dose group. **A.** Growth curve vs. standard growth curves for Japanese males,^29^ with red line indicating on-study measurements. Grey shading indicates periods of oral steroid use. **B.** Growth velocity curve vs. standard growth velocity curves for Japanese males,^30^ with red points indicating on-study measurements. Grey shading indicates periods of oral steroid use. **C.** EASI score (blue) and percent EASI score reduction (orange) by visit. **D.** Baseline characteristics. AD, atopic dermatitis; BMI, body mass index; BMI-SDS, body mass index standard deviation score; EASI, Eczema Area and Severity Index; PBO, placebo; UPA, upadacitinib.

**Fig. 2:**
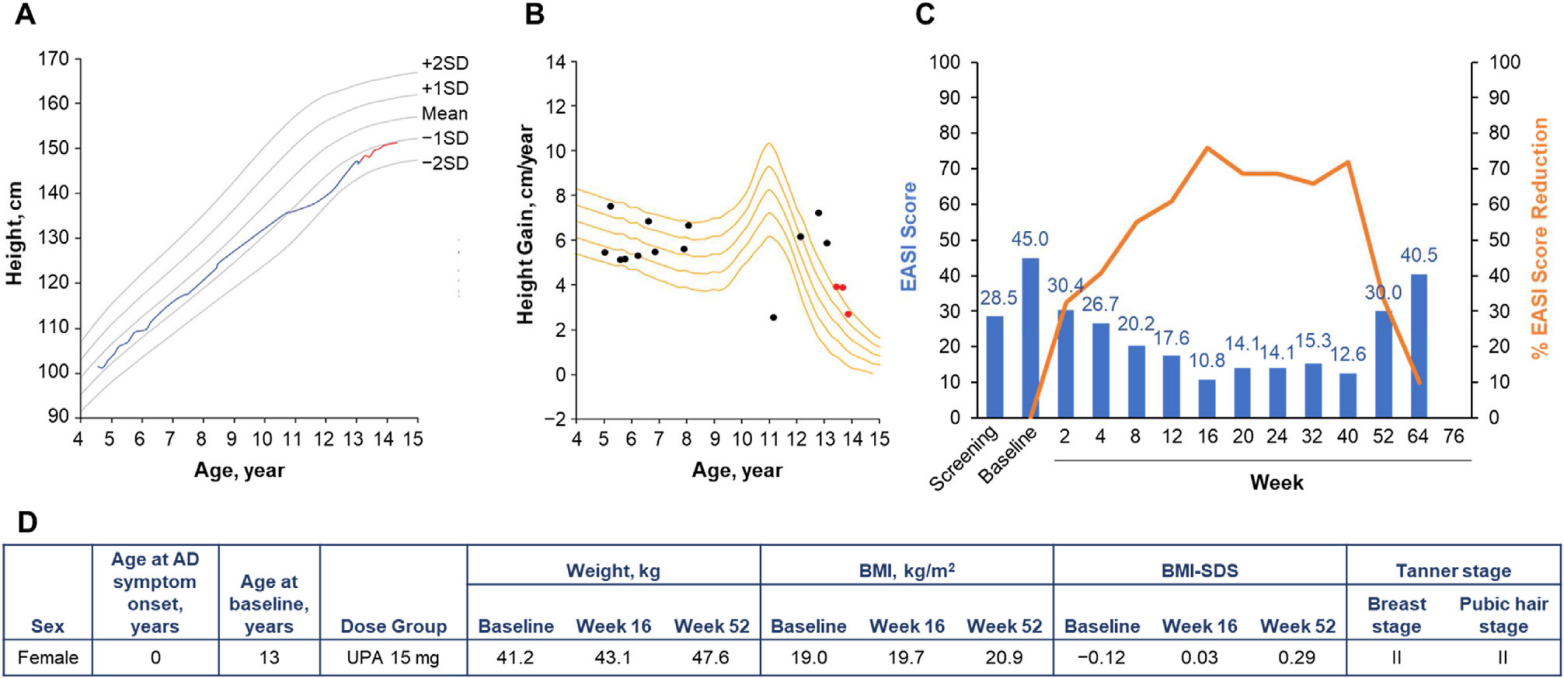
Patient 2 (female, aged 13 years) in the upadacitinib 15-mg dose group. **A.** Growth curve vs. standard growth curves for Japanese females, with red line indicating on-study measurements.^29^**B.** Growth velocity curve vs. standard growth velocity curves for Japanese females,^30^ with red points indicating on-study measurements. **C.** EASI score (blue) and percent EASI score reduction (orange) by visit. **D.** Baseline characteristics. AD, atopic dermatitis; BMI, body mass index; BMI-SDS, body mass index standard deviation score; EASI, Eczema Area and Severity Index; UPA, upadacitinib.

**Fig. 3:**
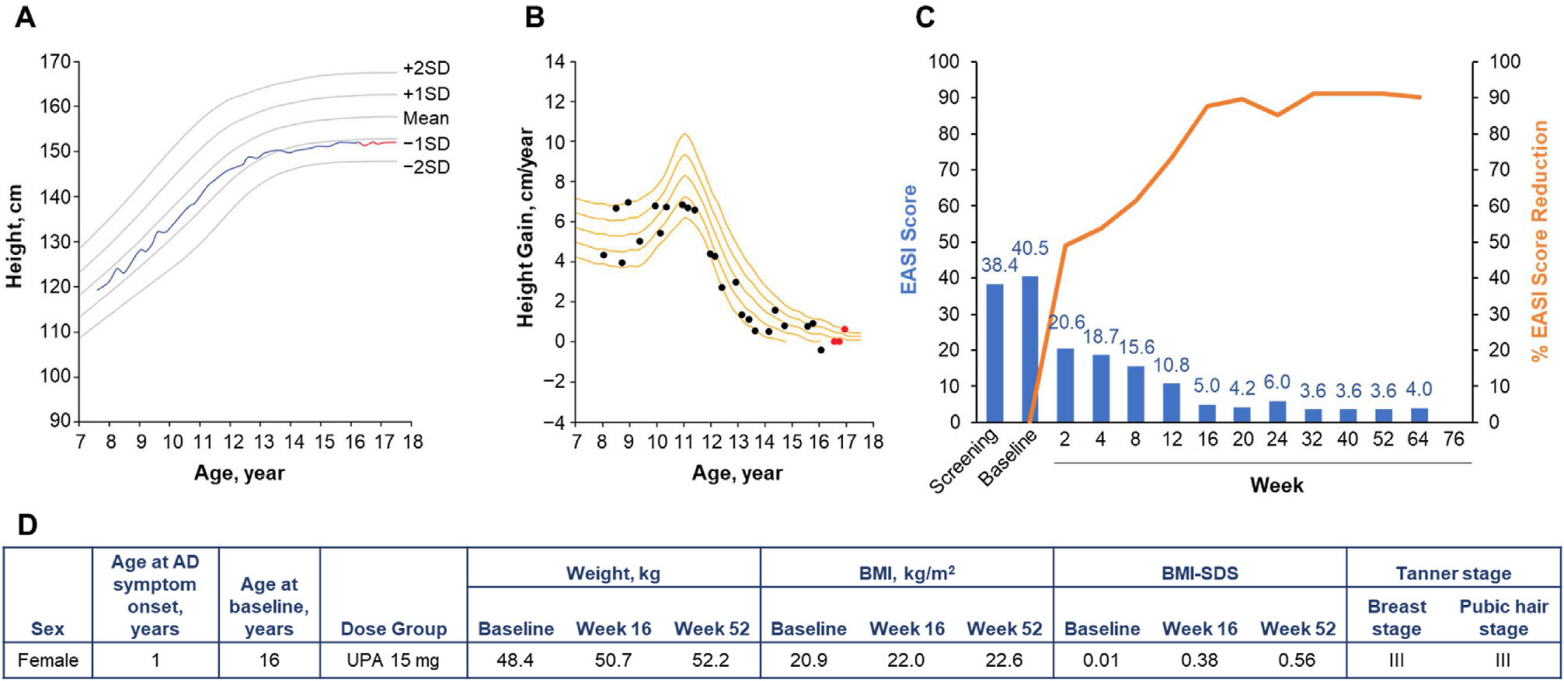
Patient 3 (female, aged 16 years) in the upadacitinib 15-mg dose group. **A.** Growth curve vs. standard growth curves for Japanese females, with red line indicating on-study measurements.^29^**B.** Growth velocity curve vs. standard growth velocity curves for Japanese females,^30^ with red points indicating on-study measurements. **C.** EASI score (blue) and percent EASI score reduction (orange) by visit **(C)**. **D.** Baseline characteristics. AD, atopic dermatitis; BMI, body mass index; BMI-SDS, body mass index standard deviation score; EASI, Eczema Area and Severity Index; UPA, upadacitinib.

**Fig. 4:**
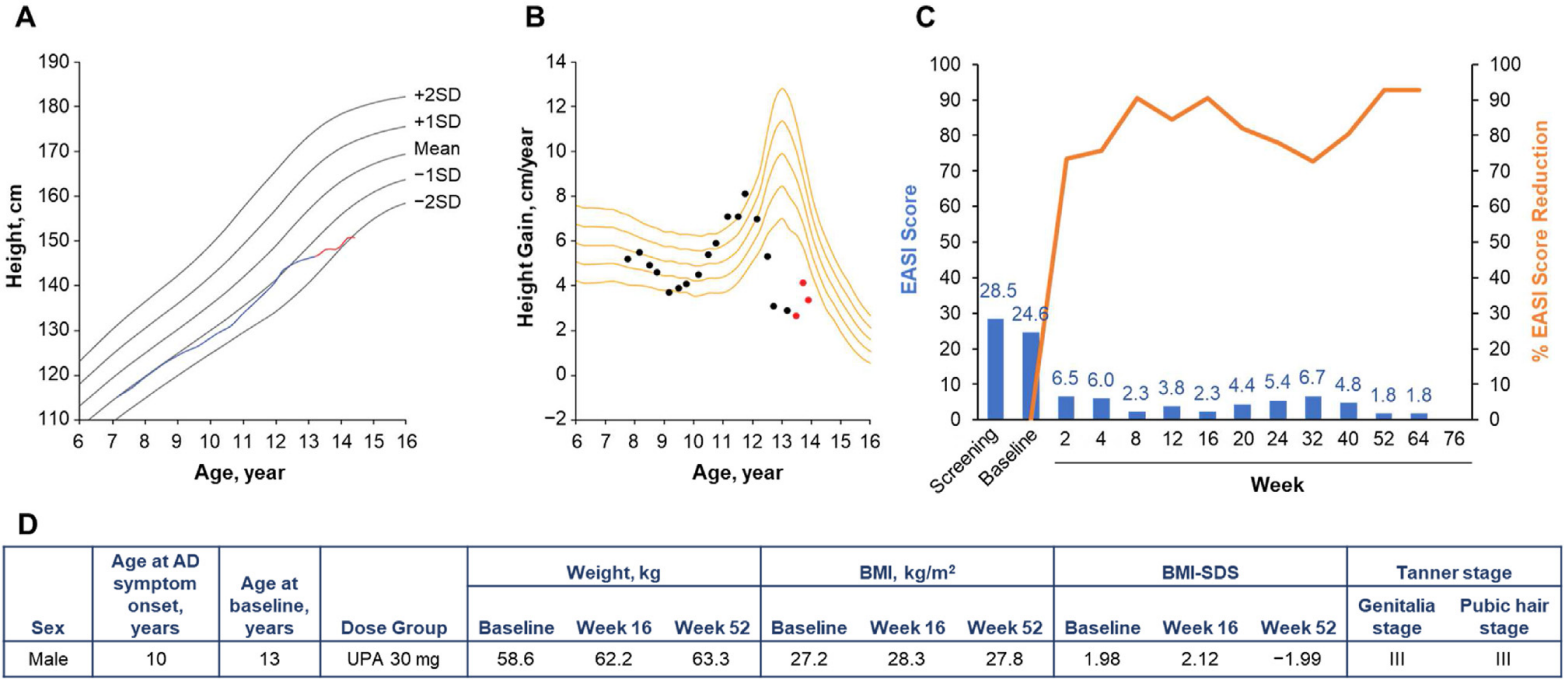
Patient 4 (male, aged 13 years) in the upadacitinib 30-mg dose group. **A.** Growth curve vs. standard growth curves for Japanese males, with red line indicating on-study measurements,^29^**B.** Growth velocity curve vs. standard growth velocity curves for Japanese males,^30^ with red points indicating on-study measurements. **C.** EASI score (blue) and percent EASI score reduction (orange) by visit. **D.** Baseline characteristics. AD, atopic dermatitis; BMI, body mass index; BMI-SDS, body mass index standard deviation score; EASI, Eczema Area and Severity Index; UPA, upadacitinib.

**Fig. 5:**
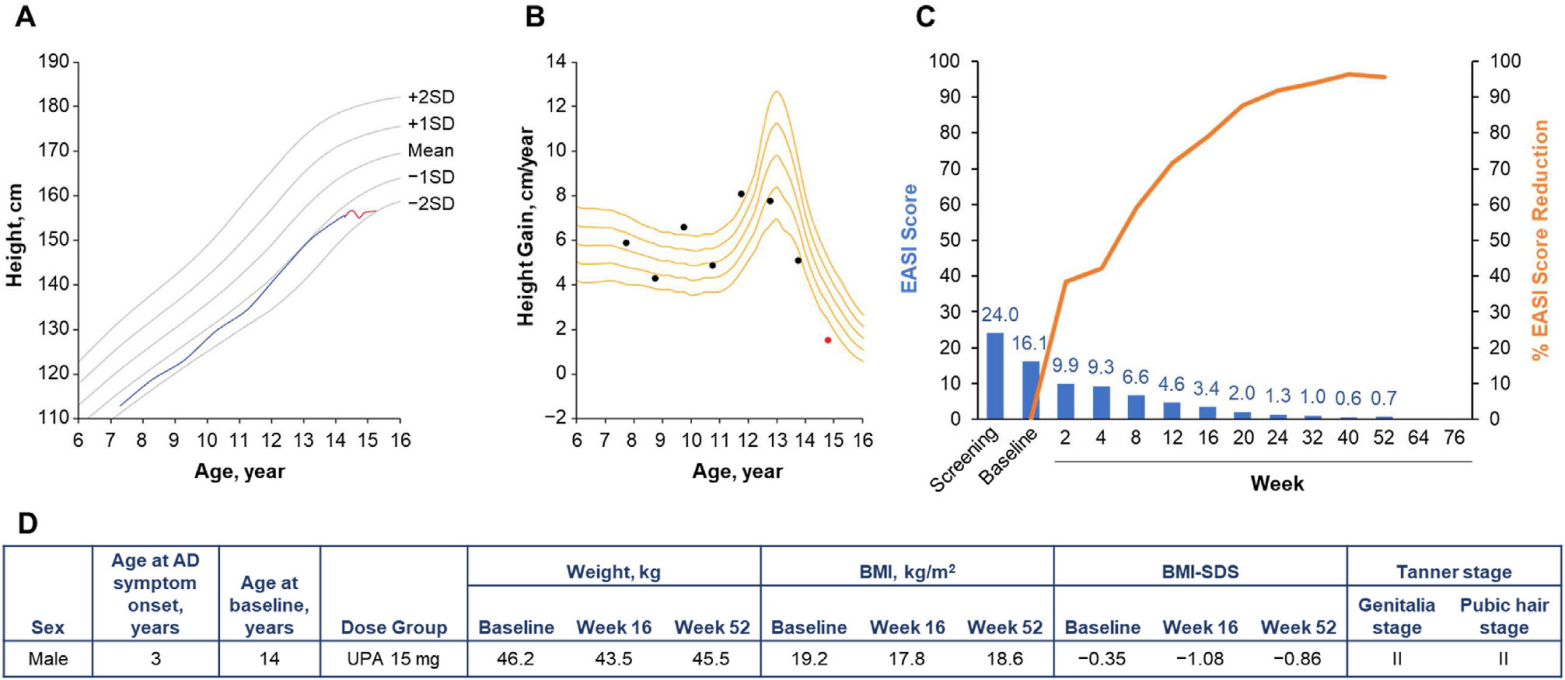
Patient 5 (male, aged 14 years) in the upadacitinib 15-mg dose group. **A.** Growth curve vs. standard growth curves for Japanese males, with red line indicating on-study measurements.^29^**B.** Growth velocity curve vs. standard growth velocity curves for Japanese males,^30^ with red point indicating on-study measurement. **C.** EASI score (blue) and percent EASI score reduction (orange) by visit. **D.** Baseline characteristics. AD, atopic dermatitis; BMI, body mass index; BMI-SDS, body mass index standard deviation score; EASI, Eczema Area and Severity Index; UPA, upadacitinib.

**Fig. 6:**
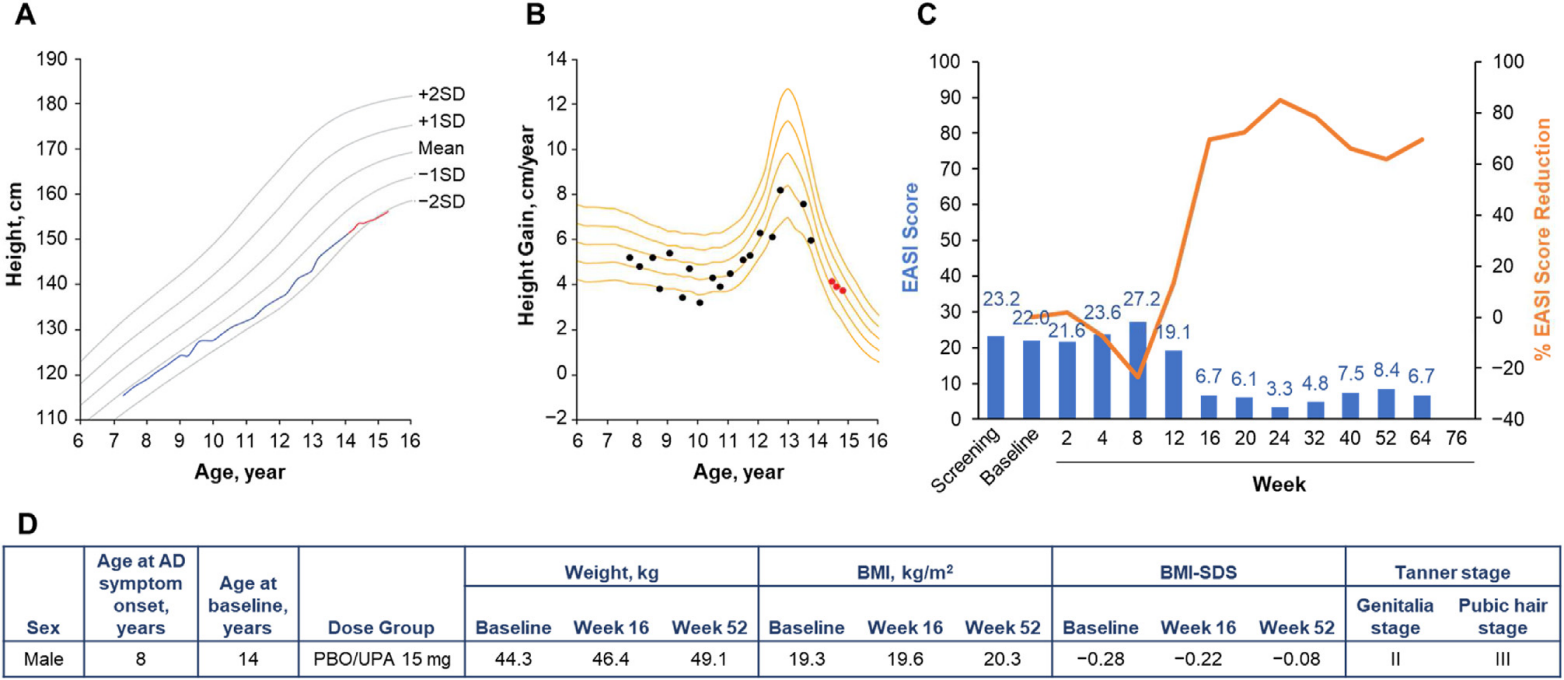
Patient 6 (male, aged 14 years) in the placebo (16-week)/upadacitinib 15-mg dose group. **A.** Growth curve vs. standard growth curves for Japanese males, with red line indicating on-study measurements.^29^**B.** Growth velocity curve vs. standard growth velocity curves for Japanese males,^30^ with red points indicating on-study measurements. **C.** EASI score (blue) and percent EASI score reduction (orange) by visit. **D.** Baseline characteristics. AD, atopic dermatitis; BMI, body mass index; BMI-SDS, body mass index standard deviation score; EASI, Eczema Area and Severity Index; PBO, placebo; UPA, upadacitinib.

Growth curves, growth velocity curves, and EASI scores by visit for the 6 adolescent patients who were in the decline phase of pubertal growth at the time of study entry are shown alongside standard growth curves for Japanese children and adolescents by sex^29^ in Fig. 1, Fig. 2, Fig. 3, Fig. 4, Fig. 5, Fig. 6. Generally, all 6 patients experienced improvements in EASI scores with upadacitinib treatment. Individual patient weights and body mass indexes were generally similar or slightly increased from baseline to week 52. Biomarkers of bone metabolism (alkaline phosphatase, calcium, and phosphate values) for all 6 patients as measured over the course of the study are shown in Fig. S1. These values remained generally stable throughout the study. No musculoskeletal adverse events or descriptions of growth abnormalities were observed in any adolescent patient.

### Individual patient cases

Patient 1 was male, aged 14 years at baseline, and was randomized to receive upadacitinib 15 mg (Fig. 1). His height was 165.7 cm at baseline and 171.4 cm at week 52. Growth velocity was low between ages 10 and 14 years, a period during which the patient was using an oral corticosteroid (betamethasone). Although the peak of growth had passed by the time of study participation, it appears that growth velocity had increased during the study period. It is likely that uncontrolled AD and/or the use of betamethasone had inhibited the peak of growth velocity and the improvement in skin symptoms and/or the discontinuation of betamethasone during the study participation may have contributed to the improvement in growth velocity. No negative impact on growth due to upadacitinib was observed.

Patient 2 was a 13-year-old female who was randomized to receive upadacitinib 15 mg (Fig. 2). Her height was 147.1 cm at baseline and 151.0 cm at week 52. Though the timing of menarche for this patient is unknown, it seems the patient's puberty started around the age of 12 judging from the growth curve, which is rather late compared with the standard Japanese adolescent growth curves.^30^ Although her growth velocity declined after trial participation, a peak (spurt) in growth velocity was observed before trial participation, indicating that she had already reached her adult height by that time. Patient 2 experienced an AD flare during the study; however, she had already reached her adult height at that time. During study participation, the patient's growth followed normal growth curve and growth velocity, which demonstrates no negative effect of upadacitinib or AD severity on growth.

Patient 3 was a 16-year-old female who was randomized to receive upadacitinib 15 mg (Fig. 3). She was 152.1 cm in height at both baseline and week 52. While this patient's growth curve appeared to be in decline during the study, review of the growth curve prior to the study and the growth velocity indicates that the patient had reached adult height prior to study participation. No negative impact on growth was observed.

Patient 4 was a 13-year-old male who was randomized to receive upadacitinib 30 mg (Fig. 4). He was 146.8 cm in height at baseline and 150.9 cm at week 52. His height increased rapidly along with weight (from 58.6 kg to 66.1 kg) and BMI (from 27.2 to 29.0) over the course of study participation (baseline to week 64). The growth spurts at ages 10 and 11 suggest that puberty was nearing its end by the time of study participation, and a further increase in height was not expected during study participation.

Patient 5 was a 14-year-old male who was randomized to receive upadacitinib 15 mg (Fig. 5). He was 155.1 cm tall at baseline and 156.6 cm tall at week 52. Although his height appeared to be low for a male aged 14–15 years, the patient appeared to enter puberty around age 11. Since a pubertal height increase of approximately 25 cm was observed prior to study entry, which is an average height gain during puberty, the patient most likely had reached adult height by the time of study participation.

Patient 6 was a 14-year-old male who was randomized to receive placebo at baseline and was rerandomized to receive upadacitinib 15 mg at week 16 (Fig. 6). His height was 151.5 cm at baseline and 155.4 cm at week 52. Because this patient participated after peak height velocity as observed in the growth velocity curve (panel B), growth velocity after participation in the clinical study was within the normal range. No negative impact on growth was observed.

## Discussion

As previously noted, the long-term use of oral corticosteroid therapies in adolescents is associated with growth impairment.^16^^,^^17^ In contrast, the potential impact of JAK inhibitors on bone growth remains unknown, and, to the best of our knowledge, no studies of growth effects of JAK inhibitors in adolescents have been reported so far. A recent study of the JAK inhibitors tofacitinib and baricitinib in animal models has identified them as bone-active drugs,^31^ with evidence suggesting that JAK inhibitors enhance Wnt signaling, thereby supporting osteoblast function. Such an effect, if present under physiological conditions, might be expected to promote bone growth. On the other hand, JAK2 is a component of activated growth hormone receptor signaling,^32^ which is significantly involved in bone growth.^33^ Inhibiting JAK2 might, therefore, be expected to retard bone growth. However, the JAK inhibitor used in the present study, upadacitinib, has a limited affinity for JAK2 relative to JAK1, its primary target.^19^

Pediatric patients with AD frequently have a higher BMI than those in the general pediatric population,^34^ which may lead to early puberty,^35^ in turn resulting in below-average adult height with decreased pubertal height gain.^36^ Among the adolescents in the Rising Up study, we suspect that some (eg, patient 4) had undergone precocious puberty, and may, therefore, have already ended their growth at a below-normal height for their age, regardless of study participation. Most adolescents had reached their adult height by the time of entry into the study.

Overall, growth curve examination of 6 patients who were in the decline phase of pubertal growth at study entry confirmed that none of the cases suggested that upadacitinib negatively affected growth among adolescent patients in this study. Patient 2 experienced a flare during the study but had already reached her adult height by that time and no further effects on growth were observed; the other 5 patients experienced substantial improvements in EASI scores during the study, suggesting that atopic dermatitis disease severity did not affect growth. Alkaline phosphatase, calcium, and phosphate concentrations were generally within the reference range for each age group and stable over the course of study participation. Elevated alkaline phosphatase levels were observed in patient 2; however, these values later normalized and the investigator had no concerns related to bone growth. In addition, no musculoskeletal adverse events indicative of bone growth abnormalities were reported in adolescents through week 52 in this study. Future studies of upadacitinib will continue to assess patient height over time and monitor adverse events related to bone growth to further inform on the impact of upadacitinib on growth in adolescents.

## Abbreviations

AD, atopic dermatitis; BMI, body mass index; BMI-SDS, body mass index standard deviation score; EASI, Eczema Area and Severity Index; PBO, placebo; TCS, topical corticosteroids; UPA, upadacitinib.

## Funding

The present study was supported by 10.13039/100006483AbbVie, Inc.

## Author contributions

TT, TS, and YO contributed to research project conception and design and data interpretation. KI contributed to research project conception and statistical analysis. JL and ART contributed to data interpretation. TT evaluated the growth curve and growth velocity curve of the patients. All authors reviewed and critiqued the manuscript throughout the editorial process and approved of the final manuscript draft submitted for publication.

## Authors’ consent for publication

All authors agree to be accountable for all aspects of the work, ensuring the accuracy and integrity of the manuscript, and consent to its publication.

## Ethics approval

This study was conducted in accordance with Good Clinical Practice Guidelines (as defined by the International Conference on Harmonisation and the Declaration of Helsinki), approved by institutional review boards at the study centers, and all parents or legal guardians of adolescent patients provided written informed consent. See details at ClinicalTrials.gov, Identifier NCT03661138. Patient anonymity was preserved using methods approved by the institutional review boards of all participating clinical trial sites.

## Authors’ consent for publication

All authors consented to the publication of this work in the *World Allergy Organization Journal*.

## Declaration of competing interests

TT has received honoraria for consultancy from AbbVie and JCR Pharmaceuticals. TS, KI, and JL are full-time employees of AbbVie, and may hold AbbVie stock or stock options. ART is a former employee of AbbVie and may hold AbbVie stock or stock options. YO has received funding/support from 10.13039/501100012030Yakult, Alcare, Kao, and Fam's Baby, and an honorarium as a speaker/consultant from 10.13039/100006483AbbVie GK, Kyorin, Maruho, 10.13039/100013995Sanofi Genzyme, Sysmex, Shiseido, Thermo Fisher, and Torii Pharmaceutical.

## Data sharing statement

AbbVie is committed to responsible data sharing regarding the clinical trials we sponsor. This includes access to anonymized individual and trial-level data (analysis data sets), as well as other information (eg, protocols and clinical study reports), as long as the trials are not part of an ongoing or planned regulatory submission. This includes requests for clinical trial data for unlicensed products and indications. These clinical trial data can be requested by any qualified researchers who engage in rigorous, independent scientific research, and will be provided following review and approval of a research proposal and statistical analysis plan and execution of a data sharing agreement. Data requests can be submitted at any time and the data will be accessible for 12 months, with possible extensions considered. For more information on the process, or to submit a request, visit the following link: https://www.abbvie.com/our-science/clinical-trials/clinical-trials-data-and-information-sharing/data-and-information-sharing-with-qualified-researchers.html.
