# Supplementary material for: Growth analysis among adolescents with moderate-to-severe atopic dermatitis receiving upadacitinib in combination with topical corticosteroids in Japan: A case study series from a phase 3, randomized, controlled trial (Rising Up)
Source: World Allergy Organ J. 2022 Sep 13;15(9):100678. doi: 10.1016/j.waojou.2022.100678 (PMC9478921; doi:10.1016/j.waojou.2022.100678)
Supplement: Multimedia component 1 [file mmc1.pdf]

SUPPLEMENTARY FIGURES

Fig. S1. Serum biomarkers of bone metabolism. Alkaline phosphatase (U/L) **(A)**, calcium (mmol/L) **(B)**, and phosphate (mmol/L) **(C)** for 6 patients who were in the decline phase of growth at study entry. Patient 1 had received oral betamethasone at times between ages 10 and 14.

Patient 1

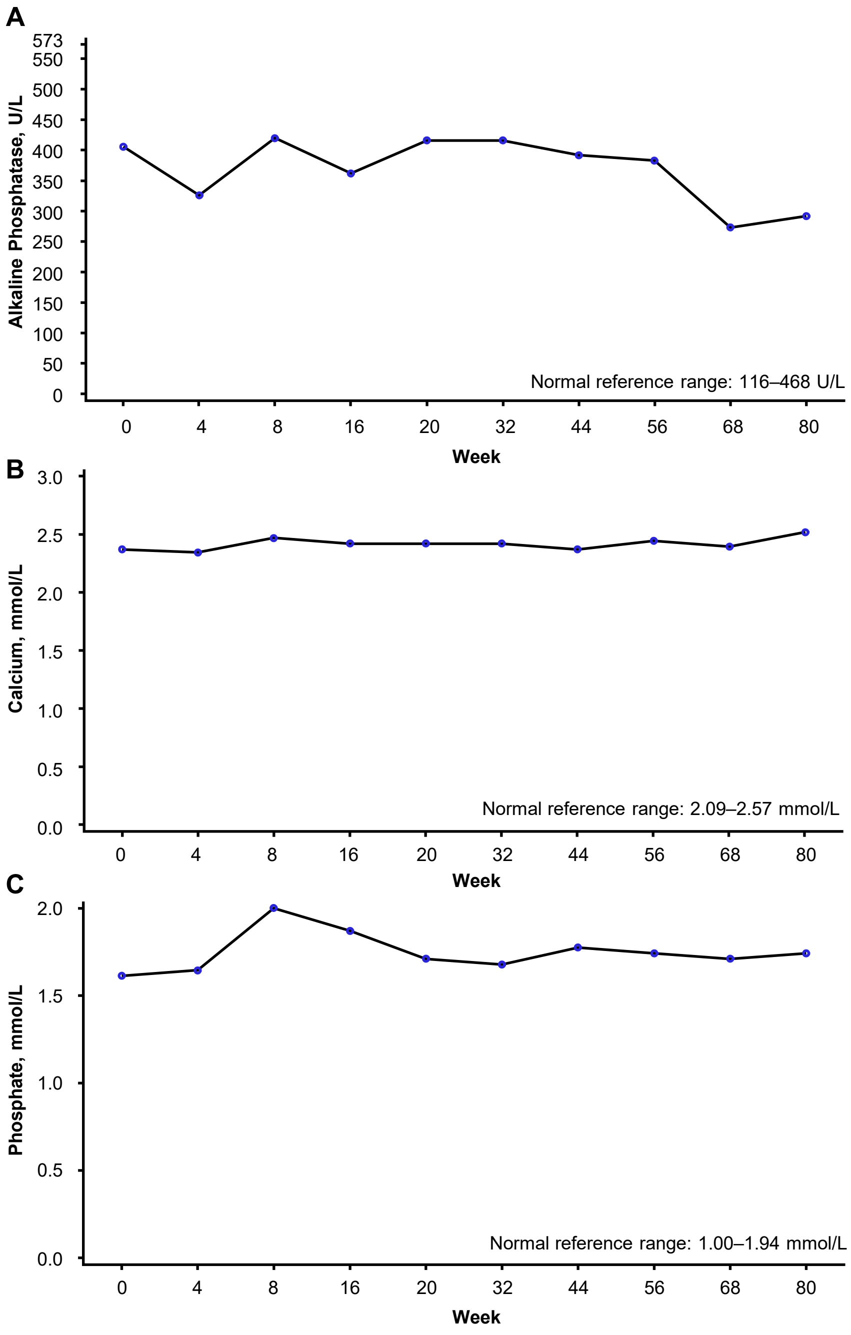
A
Alkaline Phosphatase, U/L
573
550
500
450
400
350
300
250
200
150
100
50
0
Normal reference range: 116–468 U/L
0
4
8
16
20
32
44
56
68
80
Week
B
Calcium, mmol/L
3.0
2.5
2.0
1.5
1.0
0.5
0.0
Normal reference range: 2.09–2.57 mmol/L
0
4
8
16
20
32
44
56
68
80
Week
C
Phosphate, mmol/L
2.0
1.5
1.0
0.5
0.0
Normal reference range: 1.00–1.94 mmol/L
0
4
8
16
20
32
44
56
68
80
Week

Patient 2

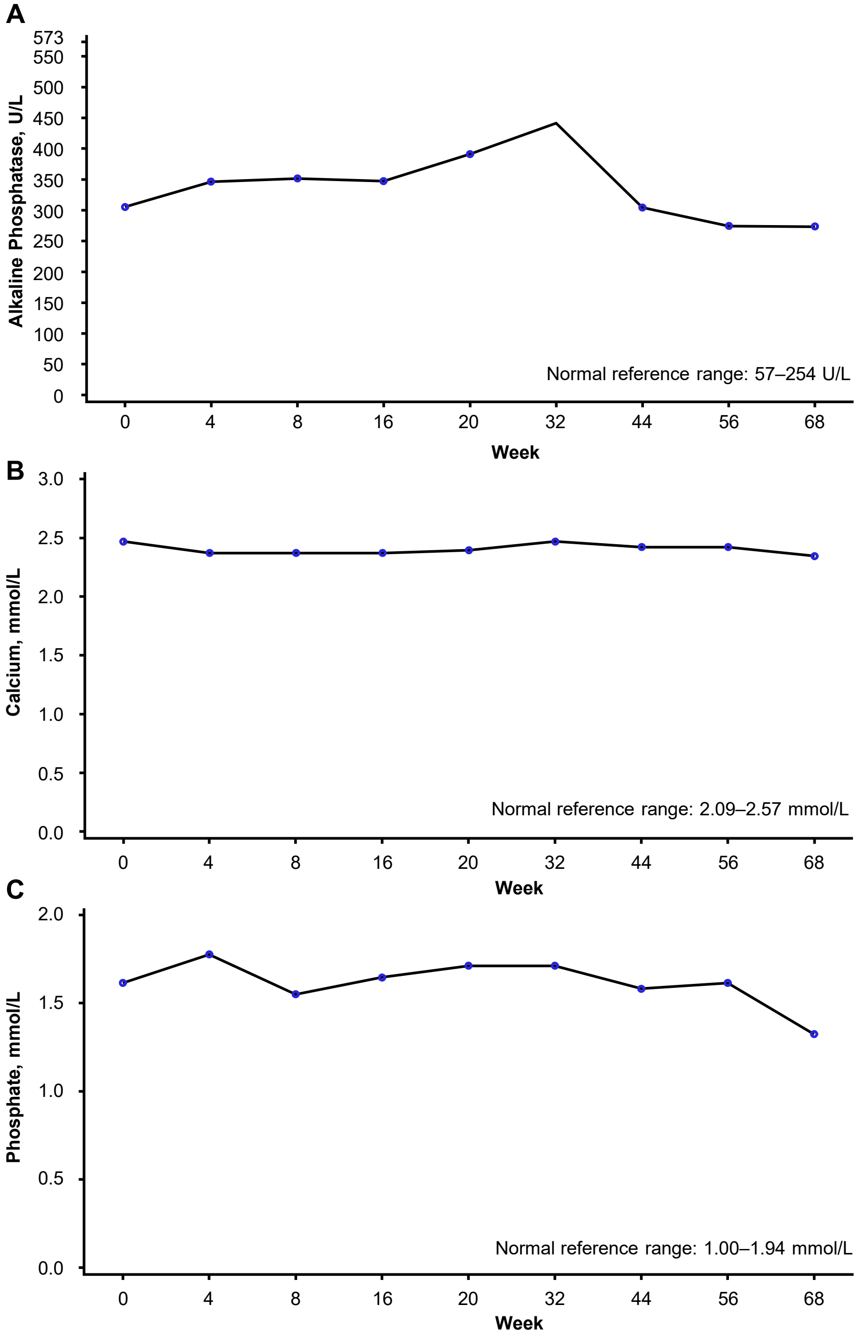
A
Alkaline Phosphatase, U/L
573
550
500
450
400
350
300
250
200
150
100
50
0
Normal reference range: 57–254 U/L
0
4
8
16
20
32
44
56
68
Week
B
Calcium, mmol/L
3.0
2.5
2.0
1.5
1.0
0.5
0.0
Normal reference range: 2.09–2.57 mmol/L
0
4
8
16
20
32
44
56
68
Week
C
Phosphate, mmol/L
2.0
1.5
1.0
0.5
0.0
Normal reference range: 1.00–1.94 mmol/L
0
4
8
16
20
32
44
56
68
Week

Patient 3

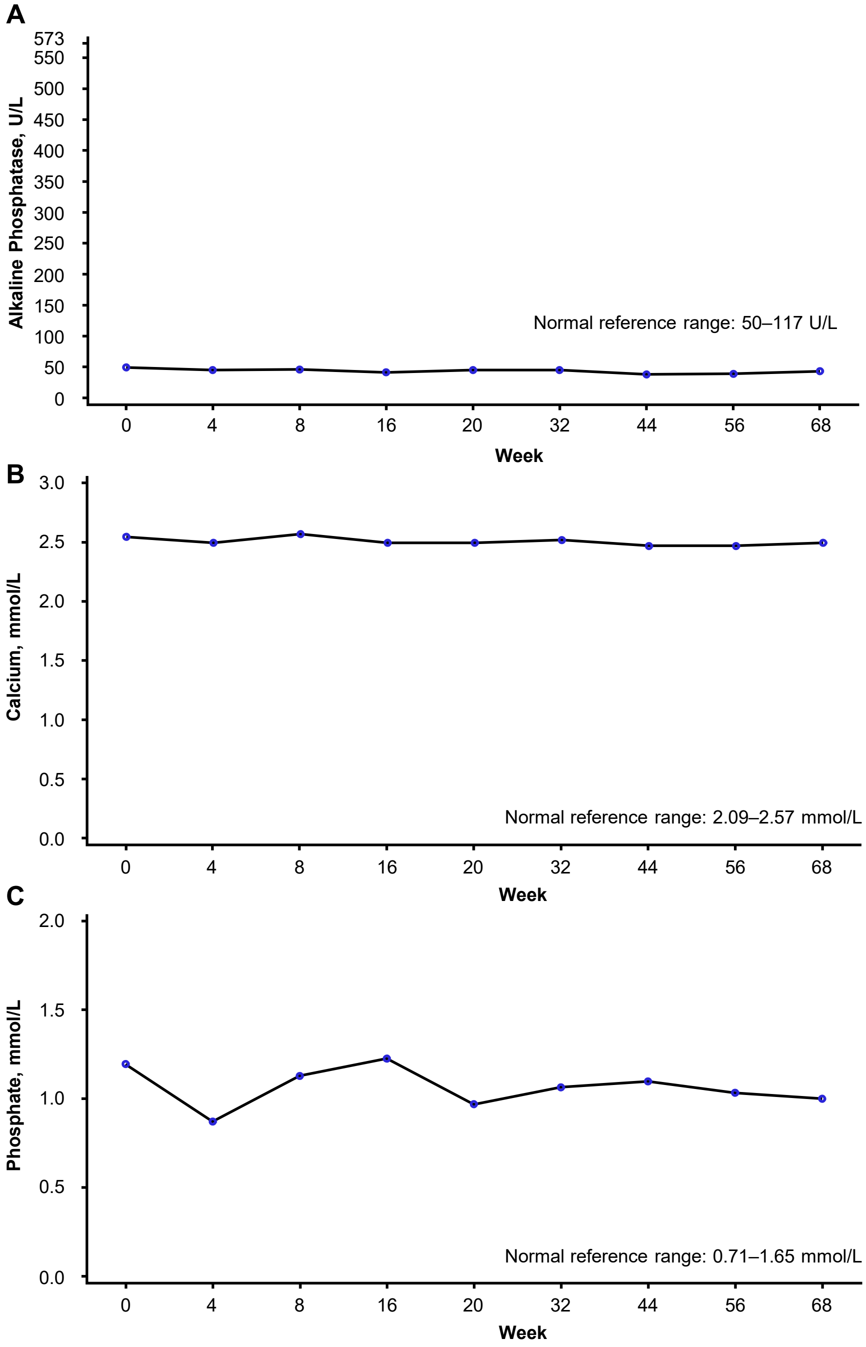

A
Alkaline Phosphatase, U/L
573
550
500
450
400
350
300
250
200
150
100
50
0
Normal reference range: 50–117 U/L
0
4
8
16
20
32
44
56
68
Week
B
Calcium, mmol/L
3.0
2.5
2.0
1.5
1.0
0.5
0.0
Normal reference range: 2.09–2.57 mmol/L
Week
C
Phosphate, mmol/L
2.0
1.5
1.0
0.5
0.0
Normal reference range: 0.71–1.65 mmol/L
Week

Patient 4

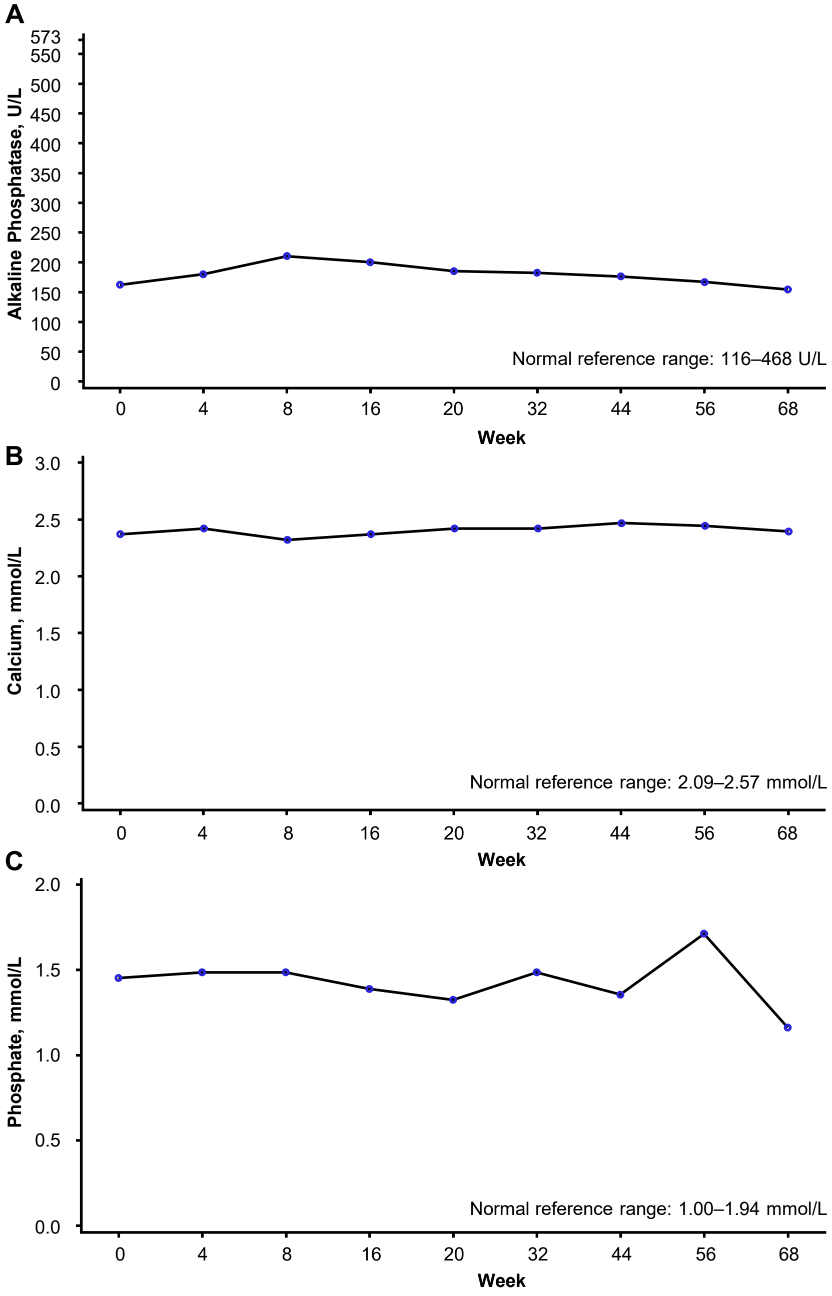

A
Alkaline Phosphatase, U/L
573
550
500
450
400
350
300
250
200
150
100
50
0
Normal reference range: 116–468 U/L
0 4 8 16 20 32 44 56 68
Week
B
Calcium, mmol/L
3.0
2.5
2.0
1.5
1.0
0.5
0.0
Normal reference range: 2.09–2.57 mmol/L
0 4 8 16 20 32 44 56 68
Week
C
Phosphate, mmol/L
2.0
1.5
1.0
0.5
0.0
Normal reference range: 1.00–1.94 mmol/L
0 4 8 16 20 32 44 56 68
Week

Patient 5

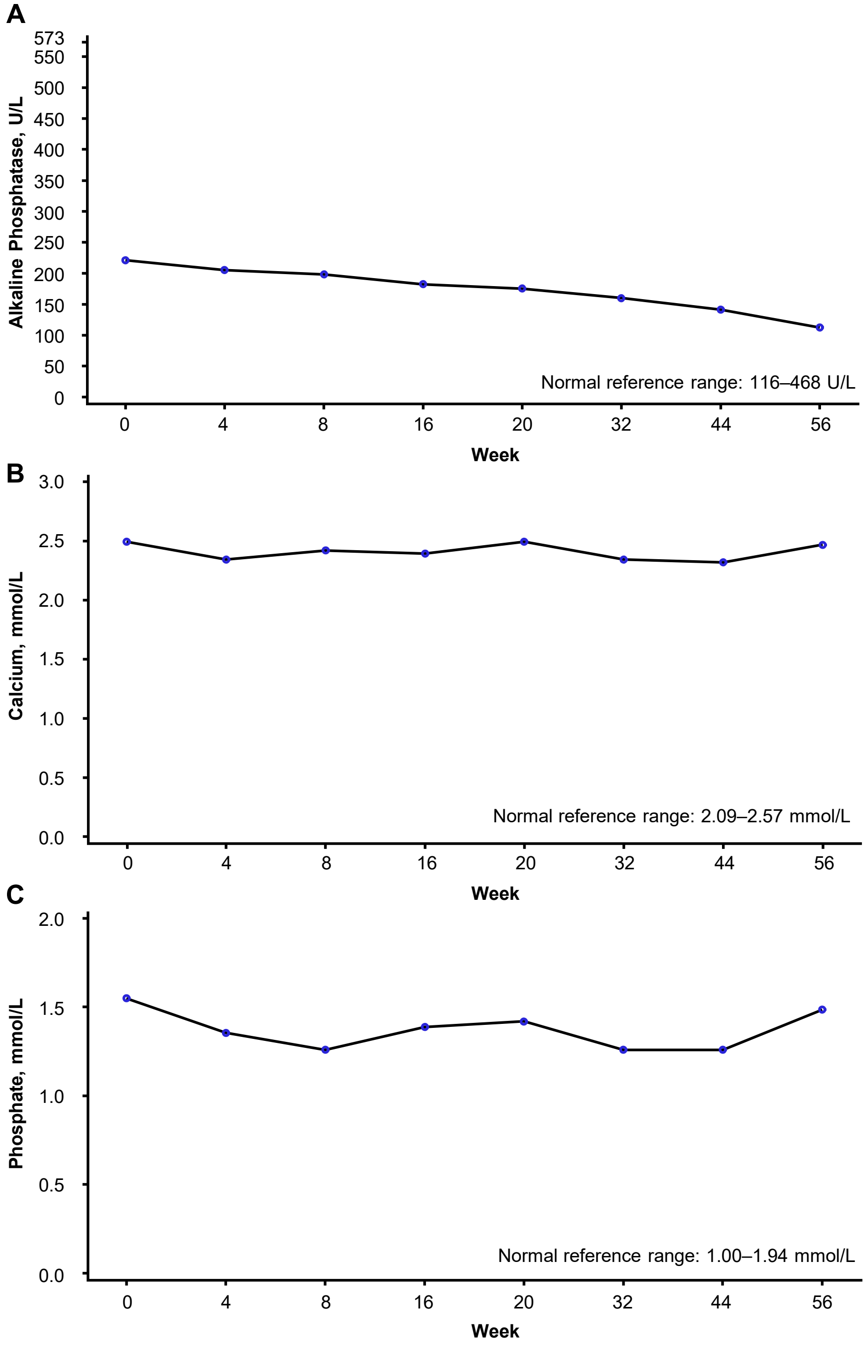
A
573
550
500
450
400
350
300
250
200
150
100
50
0
Alkaline Phosphatase, U/L
Normal reference range: 116–468 U/L
0
4
8
16
20
32
44
56
Week
B
3.0
2.5
2.0
1.5
1.0
0.5
0.0
Calcium, mmol/L
Normal reference range: 2.09–2.57 mmol/L
0
4
8
16
20
32
44
56
Week
C
2.0
1.5
1.0
0.5
0.0
Phosphate, mmol/L
Normal reference range: 1.00–1.94 mmol/L
0
4
8
16
20
32
44
56
Week

Patient 6

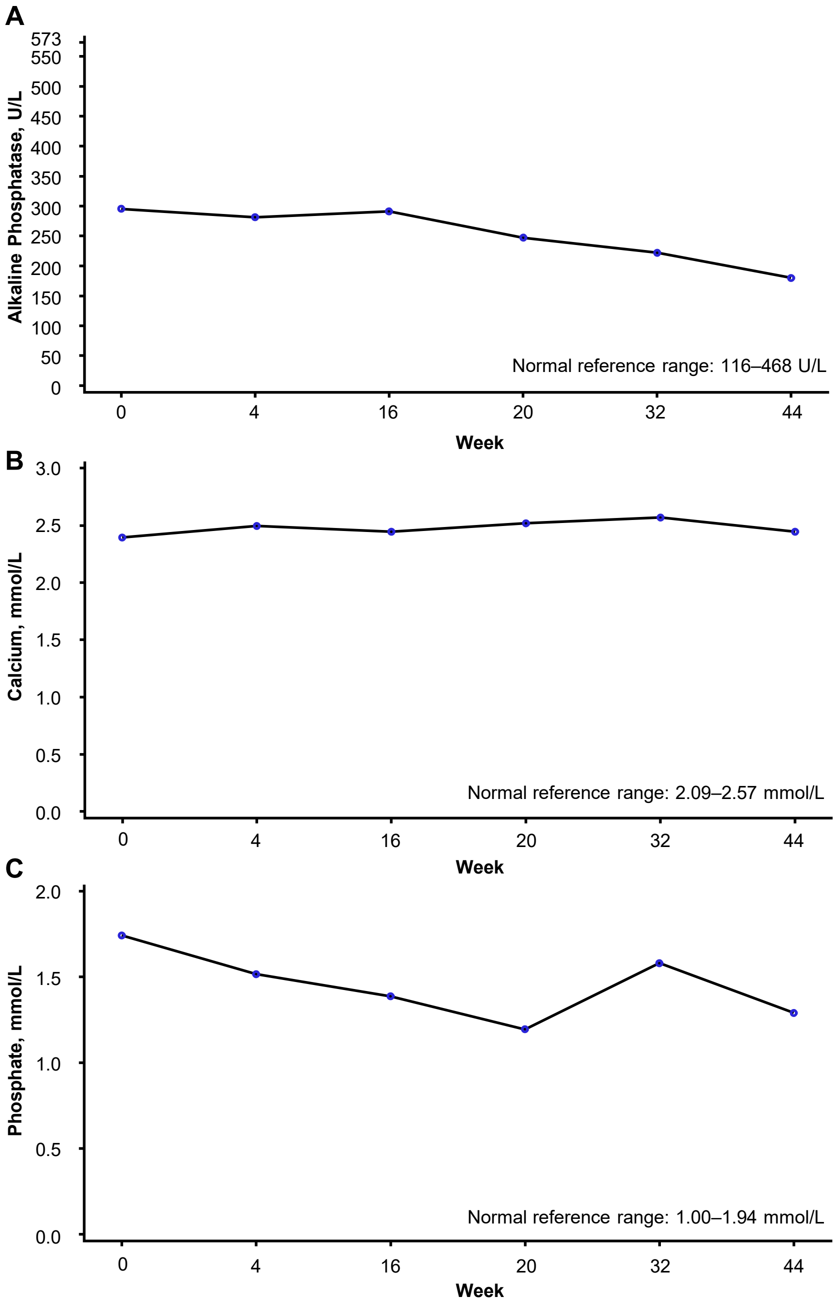
A
Alkaline Phosphatase, U/L
573
550
500
450
400
350
300
250
200
150
100
50
0
Normal reference range: 116–468 U/L
0
4
16
20
32
44
Week
B
Calcium, mmol/L
3.0
2.5
2.0
1.5
1.0
0.5
0.0
Normal reference range: 2.09–2.57 mmol/L
0
4
16
20
32
44
Week
C
Phosphate, mmol/L
2.0
1.5
1.0
0.5
0.0
Normal reference range: 1.00–1.94 mmol/L
0
4
16
20
32
44
Week
